# Reconstructing family doctors’ psychological well-being and motivation for effective performance in China: the intervening role of psychological capital

**DOI:** 10.1186/s12875-020-01182-1

**Published:** 2020-07-10

**Authors:** Xinglong Xu, Lulin Zhou, Henry Asante-Antwi, Ama Boafo-Arthur, Tehzeeb Mustafa

**Affiliations:** 1 School of Management, Jiangsu University, 301 Xuefu Road, Zhenjiang, Jiangsu P.R. China; 2 Center for Health and Public Policy Research, Jiangsu University, 301 Xuefu Road, Zhenjiang, Jiangsu P.R. China; 3 Shanghai Normal University, 2151 Gongji Road, Pudongxin, Shanghai, P.R. China; 4 School of Continuing and Distance Education, University of Ghana, P. O Box LG25, Legon, Accra, Ghana

**Keywords:** Family doctor, Psychological well-being, Motivation, Performance, China

## Abstract

**Background:**

Family practice and family doctors are critical part of China’s primary healthcare delivery in a constantly evolving society. As the first point of contact with the medical system, family practices require physically and psychologically sound and a well-motivated family doctors at all times. This is because an error can lead to loss of lives as gatekeepers of the medical system. Our study explored the extent to which positive psychological capital promotes higher performance among family doctors.

**Methods:**

A questionnaire was used to collect data from family doctors in Shanghai, Nanjing, and Beijing. We applied a structural equation analysis to analyze the causal relationship among the variables.

**Results:**

We found out that psychological well-being and job involvement significantly influences the performance of family doctors in China. The study also noted that psychological capital moderates the relationship between psychological well-being attainment, job involvement, and performance.

**Conclusions:**

Studies have shown that these pressures affect their well-being considerably. For this reason, a healthcare professional who experiences positive emotions affects the total behavior which culminates into performance.

## Background

According to Bhatnagar & Srivastava [5] health professionals (especially nurses and doctors) require good psychological and mental well-being in order to function properly. This is particularly necessary at the primary healthcare level where doctors and nurses are saddled with high workload, poor working conditions and family-related crisis that potentially threatens their mental and psychological health. Ryff et al. [29] as cited in [14] defined psychological well-being as the totality of a person’s emotional experiences and the subjective evaluation of their work and life c\ircumstances. Mental wellbeing, on the other hand, entails how well a person successfully performance a mental function that lead to productive activities, and fulfils relationships with others [14]. A person’s mental wellbeing also includes his or her capacity to adjust to changes and cope with difficulties and hardships that emerges in the course of life and work [34].

Consistent with practices across the globe, China’s primary healthcare system provides generalist clinical care and basic public health services. The objective of this system is to provide universally accessible and essential healthcare services to both individuals and families within the community as the first line of contact with the overall national health system [35]. This role requires fundamental requires continuous fundamental reform to strengthen its capacity to provide a vast network of primary care, especially to the majority of its population that lives in rural areas [18].

The current primary healthcare system in China is organized into three tiers and the intensity of care increases with a higher tier. It starts with primary care facilities in the villages and towns. Then second tier comprises of the county hospitals while the third tier includes the tertiary hospitals located in the major cities but each tier has its own challenges [36]. The first challenge relates to the number of patients. Two decades ago, the outpatients’ medical doctor in a normal hospital treated around 60–70 patients in a single day. This number has doubled in 2020 and nearly 120 patients visit a single medical doctor’s consulting room in Urumqi, Zhenjiang, Xian, Hefei, Nanning, Kunming, Nanchang, etc. every day.

The situation is worse in major cities such as Shanghai, Beijing, Nanjing, Guangzhou, Shenzhen, Hangzhou, Suzhou, etc. and these can have a significant effect on the mental and psychological wellbeing of the primary care doctor.

There are other challenges that face primary health doctors in China [35]. Generally, the healthcare industry has little room for errors but the constraints of working as a family practitioner makes doctors susceptible to errors. If these results in the loss of lives and other fatal injuries, they can significant impact on the family doctors mental and psychological well-being [6]. Gallagher [11], recommends that each primary health system must put in place appropriate coping strategies to help medical professionals to deal with the emotional, mental and psychological impacts on of their work on their well-being. Thus the psychological and mental well-being of the primary health doctor and nurse is the catalyst for their happiness, satisfaction, creative thinking, pro-social behaviour, good physical health, sense of purpose and meaning to life needed to thrive professionally despite the ups and downs of primary healthcare in China [3].

Another source of vulnerabilities of mental and psychological health faced by family care practitioners in China is that they have a very low social status, receives low salary. Compared with specialist clinicians in hospitals, family practitioners receive limited recognition for their work and this occurs in both urban and rural areas in China [19]. These conditions do not befit the status of family doctors as a special gateway to health services for families before referral to advanced medical facilities. This duty entails managing risk and uncertainty, caring for individuals in the context of their families and communities, and addressing the totality of the patient.

Yuan [39] explains that a vibrant family practice system potentially lessens the burden of work provided by specialist physicians, nurses, and other public health workers in the community health clinics in the urban areas. Through effective planning and provision of comprehensive primary health care, regardless of age or sex, continuingly, family doctors support public health services by offering health management, disease prevention, disease control, health assessment to patients. China’s initial attempt to revamp the family doctor system after the SARS virus did epidemic in 2003 did not achieve much success hence the effort to resuscitate the system in 2016. Four years on, the hope that family doctor contract services will mature and become an integral part of the primary healthcare system in China is still far from being attained.

A study conducted by Xu et al. [37] suggests that an average proportion of 60.9% of China’s family doctors intend to quit direct patient contact in the next 5 years if the current system continues. In most instances, work-related mental and psychological health issues underscore the decision to opt for other related practices. Selvaggio [32] appropriately captures thus when they allude to the fact that a more pathogenic work environment appears to be developing for a population already known to be at risk of mental and psychological ill-health including burnout, depression, and addiction. Prior studies that support this situation among China’s family doctors argue that this category of workers must abide by several rules and regulations that affect their rate of pay. Most family doctors consider the strict official guidelines given to them to follow as unpleasant interference and manipulation by people of inferior social status. In addition, family doctors in China believe that a lot of the paperwork they fill is unnecessary and tortuous.

They are subject to strict, unfriendly monitoring and evaluation requirements that are deemed unsuitable to the healthcare industry. For this reason family, doctor practice has become an unattractive field of specialization relative to medical specializations. The persistence of these challenges significantly increases the potential for mental and psychological decay among family doctors which can affect their performance. Ryff, [31] explains that psychological well-being is affected by several factors which consist of motivation, job satisfaction, self-efficacy, achievement, de-individuation, physical and psychological fatigue, environment and organization identification psychological fatigue, environment, and organization identification [17, 28].

Doctors with negative mental and p**sychological well-being** will exhibit poor mental and emotional health. This can potentially create psychological, mental, and emotional disorders that can interfere with their ability to enjoy life as primary care providers and participate as active members of the society in general. Thus psychological and mental wellbeing is the precursor to a desirable or positive work outcome which includes the sustainable performance of family doctors in China. However, Robertson & Cooper [27] assert that psychological wellbeing alone is not enough to achieve the sustainable performance of family doctors. A strand of contemporary literature in human resources for health is revisiting the concept of psychological capital and its influence on family doctor motivation particularly in the case of China.

According to Robertson & Cooper [27], positive psychology builds the human resources to be dedicated and engaged to create and guarantee the competitiveness that organizations so much desire. This position is attested in the extant literature in other. For example, Pan, Mao, Zhang, Wang, and Su [22] investigated the mediation role of psychological capital on the association between nurses’ practice environment and work engagement among Chinese male nurses and noted that creating a supportive nursing practice environment could increase male nurses’ work engagement by developing their psychological capital. They suggested that nurse managers can then provide reasonable workload and pathways for male nurses to achieve goals, thereby fostering their hope. This research on “psychological capital-work engagement-job performance” mostly focused on employees’ individual dimensions leaving the organizational dimensions.

**Fig. 1:**
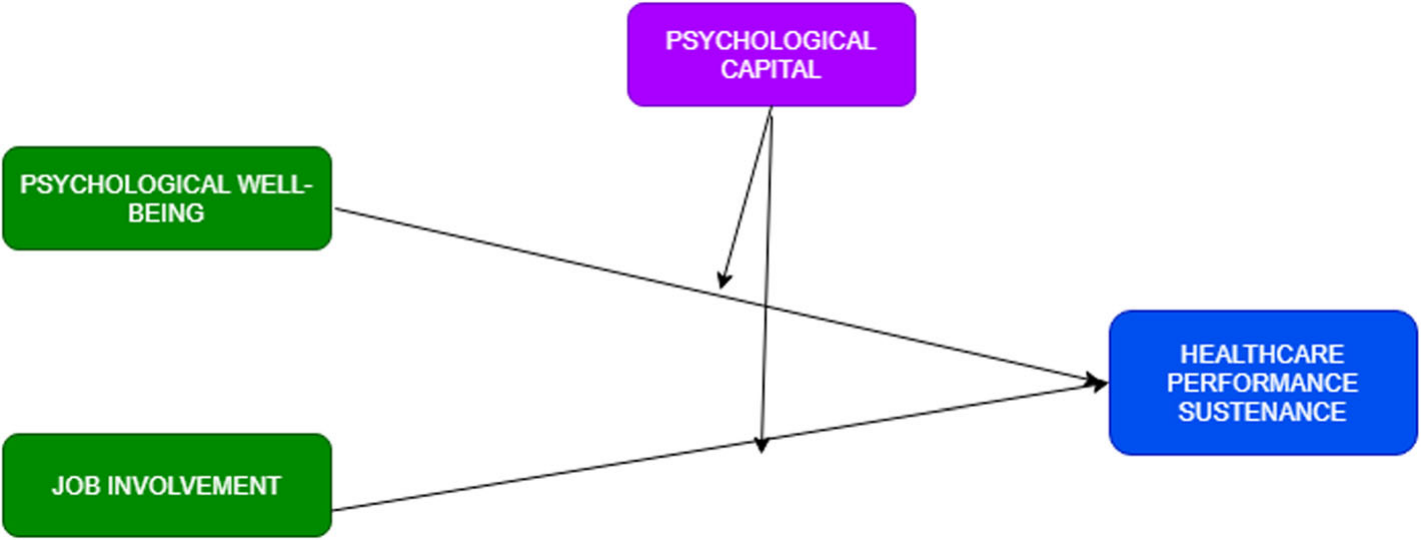
Conceptual model of the study

The work of Tien-Ming Cheng, Hong, and Yang [8] examined the moderating effects of service climate on psychological capital, work engagement, and service behavior. The study noted that attendants who showed high psychological capital tended to show more work engagement and better service behavior. The study further indicated that work engagement is the mediator between psychological capital and service behavior. Comparatively, studies have shown that there is much difference in the impact of psychological capital on government and private employees. Comparing the impact of psychological capital dimensions on the level of happiness of private and Government employees, Singh and Khan [33] found that private-sector employees had higher happiness based on higher forms of the various dimensions. The government workers had a lower level of happiness as a result of lower forms of psychological capital development; an indication that an enhanced psychological capital significantly influences employees’ level of happiness which subsequently impact their well-being. The current crisis in family doctor motivation in China offers an opportunity to explore the intermediating role of psychological capital in the relationship between psychological wellbeing and job involvement on one hand and sustainable performance of family doctors. This is because, in the traditional human resource strategy literature, psychological well-being is conceptualized to mean the experience of positive emotions that is global than negative emotions. Although most studies continue to concentrate on the negative emotions, Lazarus [16] new findings emanating from the proponents of positive psychology point to the need to advance argument on how positive emotions influence the total functioning of the individual, [10]. In this paper, we apply a novel structural equation model to explore effect of interplay between psychological wellbeing, psychological capital and job involvement on family doctor motivation in Shanghai, Nanjing and Beijing in China. First we explain the methods used in the study and present our analytical procedure and results. Next we discuss the findings and make our conclusions. Figure 1 shows the final conceptual model for the current study.

## Methods

### Study design and sample

A quantitative research study was designed to explore the direct influence of the psychological wellbeing of family doctors and job involvement (independent variables) on family doctor’s performance (dependent variable). Secondly the study also explored the moderating effect of psychological capital (hope, the self-efficacy, the optimism, the resilience) on the two direct relationships. This cross-sectional study was conducted among practicing family doctors, potential family doctors and past family doctors selected in Shanghai, Beijing, and Nanjing cities between July 2019 and December 2019. These cities were chosen because family doctor contract services are relatively developed in Chinese megacities than in other parts of China. An initial number of 780 questionnaires were designed and sent to family doctors who were randomly selected by cluster sampling to accommodate the three different groups of targeted doctors. The number of years of work experience of the doctors was a major factor in deciding a qualified participant. A target least 5 years post-qualification experience was deemed sufficient. A similar effort was put into recruiting both male and female participants to compare responses were necessary. A self-administered questionnaire was directly administered to them after obtaining their written consent.

A questionnaire was given to each worker that contained a declaration of anonymity, information secrecy, voluntary participation, and voluntary withdrawal. The co-investigators directly distributed, retrieved, codded, and managed the questionnaires, and where possible respondents were provided with an explanation without inducement for any unclear questionnaire items. Items in the questionnaire were based on appropriate items in pre-existing measurement scales and respondents needed between 10 and 1 5 min to complete a full questionnaire. At the end of the research period, 108 questionnaires were not returned and 81 questionnaires with missing data were regarded invalid. Thus effective response was made up of 591 respondents which represent a response rate of approximately 76%. The study was approved by the Ethics Committee of Centre for Health and Public Policy Research and validated by the Human Experimentation of Jiangsu University in China.

### Measurement of psychological wellbeing

Psychological wellbeing was measured using the shortened version of the psychological wellbeing (PWB) scale developed by Ryff [30]. This model measures six aspects of psychological wellbeing and happiness namely; autonomy, environmental mastery, personal growth, positive relations with others, purpose in life, and self-acceptance (Ryff et al., [29]; adapted from [30]). The 18 item version is configured with a 7 point Likert scale where (1 = strongly agree; 7 = strongly disagree). Response to the individual questions is composed of six subscales and a composite value of the 6 subscales is used as a proxy score of psychological wellbeing of each doctor.

### Measurement of psychological capital

The Psychological Capital Questionnaire (PCQ) was used in measuring Psychological capital (PsyCap). It is the most widely used standard scale and has been validated in a different research context. This questionnaire was developed by [20] and consists of 24 items composed into four subscales namely hope, optimism, resilience, and self-efficacy. The scale is configured on a six-point Likert scale measure ranging from 1 (‘strongly disagree’) to 6 (‘strongly agree’).

### Measurement of job involvement

Kanungo’s [13] job involvement scale was used to measure the concept of job involvement. The model is a 10-item Job Involvement Questionnaire. This questionnaire was adopted due to its high internal consistency and wide applicability in a different research context. The response scale to this questionnaire is on a 10-point Likert scale ranging between “do not agree/not applicable to me” to “fully agree/fully applicable.

### Measurement of performance sustenance

Performance sustenance, on the other hand, was measured based on four constructs which include frequency of patients’ visits [36], frequency of patients complain [7], intention to stay or re-enrol in family practice [19], doctors days worked [38]. The response scale for items measured was (5 = Strongly Agree to 1 = Strongly Disagree). The composite value was determined and used as a proxy for performance sustenance.

### Analytical model

Firstly descriptive statistics were computed using SPSS and inferential statistics was conducted using AMOS version 22**.** The Pearson moment correlation coefficient was conducted to determine the significance and possible multicolinearity in the relationship between the variables. Subsequently, a mixed structural equation model was modelled to establish the direct effect of family doctors’ psychological wellbeing and involvement on performance sustenance. Similar model was done to determine the moderating effect of psychological capital on the direct relationships. A structural equation model is a better analytical model for this research because it produces more robust inferences compared to the traditional regression model. A structural equation helps to analyse the influence of predictor variables on different dependent variables simultaneously. The specific model used in our study is expressed in a matrix form as follows:

$$ \left[\begin{array}{c}{\mathrm{y}}_1\\ {}{\mathrm{y}}_2\\ {}\Lambda \\ {}{\mathrm{y}}_{\mathrm{p}}\end{array}\right]\kern0.62em =\kern0.62em \left[\begin{array}{cccc}0& {\upbeta}_{12}& \Lambda & {\upbeta}_{1\mathrm{p}}\\ {}{\upbeta}_{21}& 0& \Lambda & {\upbeta}_{2\mathrm{p}}\\ {}\Lambda & \Lambda & \Lambda & \Lambda \\ {}{\upbeta}_{\mathrm{p}1}& {\upbeta}_{\mathrm{p}2}& \Lambda & 0\end{array}\right]\kern0.62em \left[\begin{array}{c}{\mathrm{y}}_1\\ {}{\mathrm{y}}_2\\ {}\Lambda \\ {}{\mathrm{y}}_{\mathrm{p}}\end{array}\right]\kern1em +\kern1em \left[\begin{array}{cccc}{\upgamma}_{11}& {\upgamma}_{12}& \Lambda & {\upgamma}_{1\mathrm{q}}\\ {}{\upgamma}_{21}& {\upgamma}_{22}& \Lambda & {\upgamma}_{2\mathrm{q}}\\ {}\Lambda & \Lambda & \Lambda & \Lambda \\ {}{\upgamma}_{\mathrm{p}1}& {\upgamma}_{\mathrm{p}2}& \Lambda & {\upgamma}_{\mathrm{p}\mathrm{q}}\end{array}\right]\kern0.62em \left[\begin{array}{c}{\mathrm{x}}_1\\ {}{\mathrm{x}}_2\\ {}\Lambda \\ {}{\mathrm{x}}_{\mathrm{q}}\end{array}\right]\kern1em +\kern1em \left[\begin{array}{c}{\upzeta}_1\\ {}{\upzeta}_2\\ {}\Lambda \\ {}{\upzeta}_{\mathrm{p}}\end{array}\right] $$

1 $$ \mathbf{y}=\mathbf{By}+\Gamma \mathbf{x}+\boldsymbol{\upzeta} $$

where for each of the causal parameters (psychological wellbeing and job involvement), the γ’s and the β’s, the subscripts follow the same pattern. In the matrix, the *p* by *p* B matrix holds the coefficient of regression of the *y* variables whereas the other *y* variable with 0’s on the diagonal indicates that a variable cannot cause itself. The model also contains the *p* by *q* Γmatrix which contains the coefficients of the *y*’s on the *x*’s. The error vector (errors-in-equations or specification errors) ζ, is p by 1. For example, we assumed that E(y) = 0 and E(x) = 0 and these have absolutely no impact on both the variance and the covariances of the variables if we assume the independence of *x* and ζ. Finally, we adopted a second-order factor model that assumes that the factors can form higher-order factors. We ensured that the correlation among the factors has the correct structure as a result of the latent variables. This leads to the following path diagram indicated below;

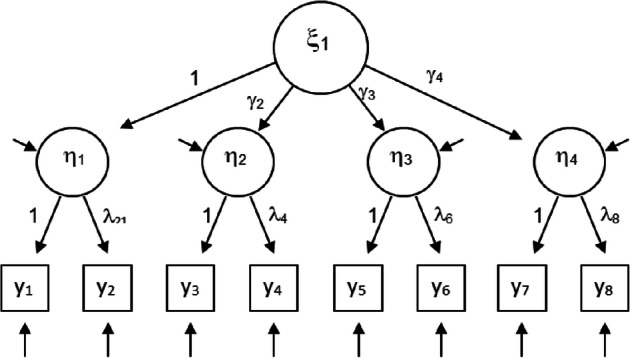



(Adopted from Bediako et al., [4])

In this model each η has a unique factor and a known variable while ξ_1_ represents the higher-order factor. Generally, we write the second-order factor analysis of the mixed structural equation as follows;

2 $$ y={\Lambda}_y+\varepsilon $$

3 $$ \upeta =\Gamma \upxi +\upzeta $$

This special case of an SEM with latent variables can be rewritten more compactly as

4 $$ \mathbf{y}={\Lambda}_y\left[\Gamma \upxi +\upzeta \right] $$

In this model, it is assumed that Cov(ε, ζ) ***=*** 0, Cov(ζ, ζ) ***=*** 0, V(ε) **=** Θε, V(ζ) **=** Ψ and V(ζ) **=** Φ**.**

In this model also, the variance of y, **Σ**, assumes a unique aesthetic form mathematically expressed as follows;

5 $$ V\left(\mathbf{y}\right)={\boldsymbol{\Lambda}}_y\left[\boldsymbol{\Gamma} \boldsymbol{\Phi} \boldsymbol{\Gamma} \hbox{'}+\boldsymbol{\Psi} \right]\boldsymbol{\Lambda} {\hbox{'}}_y+{\boldsymbol{\Theta}}_{\varepsilon } $$

where [**ΓΦΓ** '  **+ Ψ**] represents V(**η**). (6)

Next, we used summed indicators to “specify” the latent variables (psychological wellbeing and job involvement) and the regression model for the structural equation used the “no origin” option.

Consistent with prior works of Rabenu et al. [26], we set a strict level of significance for the regression coefficients (95% confidence interval) for each latent variable. We accordingly evaluated the reliability, validity and internal consistency of each latent variable using appropriate respected techniques and each latent variable met the criteria based on Pallant et al’s [21] minimum threshold. Next, the single-construct measurement model (MM) for each latent variable was evaluated to ensure it fitted the data. The reliability, validity, and internal consistency of the latent variables were evaluated and subsequently averaged to obtain the error attenuated covariance matrix (CN). Using the procedure set out in Ping [24], the resultant matrix was adjusted to obtain the measurement errors. The resulting Error-Adjusted (Err-Adj) CM was used to estimate eqs. 1 and 2 without omitting the variables. This approach is much superior to existing approaches because a measurement error is determined by measuring model loadings and measurement error variances from the “no dummies”. In this instance using the measurement model loadings and measurement error variances from the “no dummies” for Eq. 1 was applied. Related studies that have used this approach include; Anderson et al., [1]; Asante-Antwi, Zhangxiao Tian et al. [2], Kong, Akomeah et al. [15]. Next, the Err-Adj CM was used as inputs into the least square structural regression model. Again Ping [25] as cited in Kong, Akomeah et al. [15] judges this approach to be a much superior method of validating the integrity of a data relative to other traditional options since it produces fairly accurate and consistent structural coefficients in a model. Finally, we inputted the parameter estimates obtained from the no dummies MM into the latent variable regression EXCEL spreadsheet to produce the Err-Adj CM matrix based on the following computations;

7 $$ \mathrm{Var}\left({\upxi}_{\mathrm{x}}\right)=\left(\mathrm{Var}\left(\mathrm{X}\right)-{\uptheta}_x\right)/{\Lambda \mathrm{x}}^2 $$

8 $$ \mathrm{Cov}\left({\upxi}_{\mathrm{x}}{\upxi}_{\mathrm{z}}\right)=\Big(\mathrm{Cov}\left(\mathrm{X},\mathrm{Z}\right)/{\Lambda}_{\mathrm{x}}{\Lambda}_{\mathrm{z}} $$

where *Var*(*ξ*_*x*_) represents the expected error-adjusted variance of the regression inputs *x, Var*(*X*) represents the error attenuated variance of *x*.

Λ_x_ represents the average λ_X1_ + λ_X2_ + λ_X3_ … λ_xn_), where

*avg* (*θ*_*x*_) *= Var*(*ε*_*x*1_) *+ Var*(*ε*_*x*2_) *+ Var*(*ε*_*x*3_) *… + Var*(*ε*_*xn*_)*,* in which case the ε_X_’s and the λ’s represent the measurement error and the measurement model respectively. The *Cov*(*ξ*_*x*_*ξ*_*z*_) represents the desirable level of error-adjusted covariance of *x* and *z*, and *Cov* (*x*, *z*) represents the error attenuated covariance of *x* and *z*,

Finally, we inputted the *Err-Adj CN* into the regression function based on the “regression-through-the-origin” option. As recommended in Warren, White et al. as cited in Bediako et al. [4], we corrected the coefficient standard error *(SEs)* i.e. the SEs of *β*_1_, *β*_2_, … ... in eq. *(6)* produced by the Err-Adj for measurement error by adjusting the *SE* from regression using the Root Mean Square Error *(RMSE)* as expressed as follows;

9 $$ \mathrm{RMSE}=\sqrt{\frac{1}{n}\sum \limits_{i=1}^n{\left({y}_i-{\hat{y}}_i\right)}^2} $$

where *y*_*i*_ represents the observed and ${\hat{y}}_i$ represents the predicted values based on the Err-Adj CM [22, 23]. This implies that the correct Standard Errors for the Err-Adj CM structural coefficients involve the Standard Error from the regression based on the err-unadj CM. This leads to the formulation of the ratio of the standard error (SE) from err-unadj CM regression and the standard error (SE) from Err-Adj CM regression which is formulated as;

10 $$ {SE}_A={SE}_U\times \frac{RMSE_U}{RMSE} $$

in which case *SE*_*A* A_ is the Err-Adj CM regression standard error, *SE*_*U*_ is the standard error obtained from the err-unadj CM regression, RMSE_U_ is the standard error obtained from the err-unadj CM regression, and RMSE_A_ is the standard error obtained from the err-unadj CM regression.

## Results

### Descriptive statistics

Table 1 shows the inter-correlation matrix between the latent variables as well as the relationship between the latent variables and the moderator and dependent variables. The results indicates that psychological well-being was positively and significantly correlated with psychological capital (r = 0.45) and performance (r = 0.55) but not job involvement. This means that an increase in the psychological wellbeing of a family doctor is positively associated with their performance and their psychological capital. This also supports the absence of multicolinearity among the independent variables (psychological wellbeing and job involvement). Similarly, a higher job involvement is positively associated with psychological capital (r = 0.55) and performance (r = 0.44). Finally, the analysis also shows that psychological capital is positively associated with performance (r = 0.54) and this is statistically significant at a 95% confidence interval.

**Table 1:** Correlation, mean and standard deviation

Variable	Mean	SD	1	2	3	4
**1**.Psychological Capital	16.5	11.7	_			
**2**.Psychological Well-being	11.8	6.3	.45^a^	_		
**3**. Job Involvement	4.6	4.3	.34^a^	. 44^b^	_	
**4.** Performance Sustenance	12.1	6.1	.55^a^	.47^a^	.54^a^	_

^a^ Correlation is significant at the 0.01 level (2-tailed) at the diagonal

^b^ Correlation is significant at the 0.05 level (2-tailed)

### Reliability and validity test

Confirmatory factor analysis (CFA) was adopted to assess the adequacy of the measurement components. Reliability and validity of the constructs was also assessed and showed that the item-to total correlations were stronger. Some scholars have argued that factor loadings from .50 should be an acceptable loading. For this reason, the constructs that reported standardized factor loadings above the .50 criteria were accepted as recommended by Hair et al. [12]. Based on this threshold, all, the measures of the study were considered as showing satisfactory reliability. Again all the constructs showed high average variance extracted (AVE) and that further strengthens the evidence of strong evidence of convergent validity. The results of CFA, reliability, and convergent validity are shown in Table 2

**Table 2:** CFA, reliability and validity index

Observed First-Order and Second-Order Latent Variables χ2 = 141.284, df = 61, χ2/df = 2.316 TLI = .988, CFI = .982, NFI = .955, IFI = .993, RFI = .965 RMSEA = .035				
VARIABLE	α	CR	AVE	FACTOR LOADING
**Psychological Capital (PSYCAP)**	**.911**	**.687**	**.929**	
Hope	.786	.920	.746	.934
H1				.892
H2				.923
H3				.781
Self-Efficacy	.916	.942	.729	.942
SE1				.763
SE2				.861
SE3				.882
SE4				.854
SE5				.811
Optimism	.907	.939	.720	.963
OPM1				.852
OPM2				.844
OPM3				.871
OPM4				.802
OPM5				.743
Resilience	.923	.984	.751	.987
RS1				.811
RS2				.863
RS3				.854
RS4				.842
RS5				.833
**Job Involvement**	**.787**	**.904**	**.759**	
JI3				.784
JI6				.901
JI7				.923
**Psychological Well-being**	**.911**	**.929**	**.687**	
PWB5				.763
PWB6				.882
PWB7				.851
PWB8				.884
PWB9				.801
PWB10				.784
**Performance**	**795**	**.848**	**.582**	
JP1				.764
JP2				.781
JP3				.734
JP4				.773

### Confirmatory factor analysis

Confirmatory factor analysis (CFA) is an important statistical tool for probing the nature of relationships among latent constructs. It is important because it helps to measure the construct validity, identify method effects, and helps in evaluating the factor invariance through time and groups. The use of Confirmatory Factor Analysis (CFA) continues to gain ground in the psychological literature as a result of the belief researchers have in the Structural Equation Model as a robust model specifically. Given the key impact CFA makes in the measure development and due to the understanding that having a tool that manages the measurement of variables effectively, it can be presumed to be paramount quantitatively simply because its role is crucial to the results a researcher reports

**Fig. 2:**
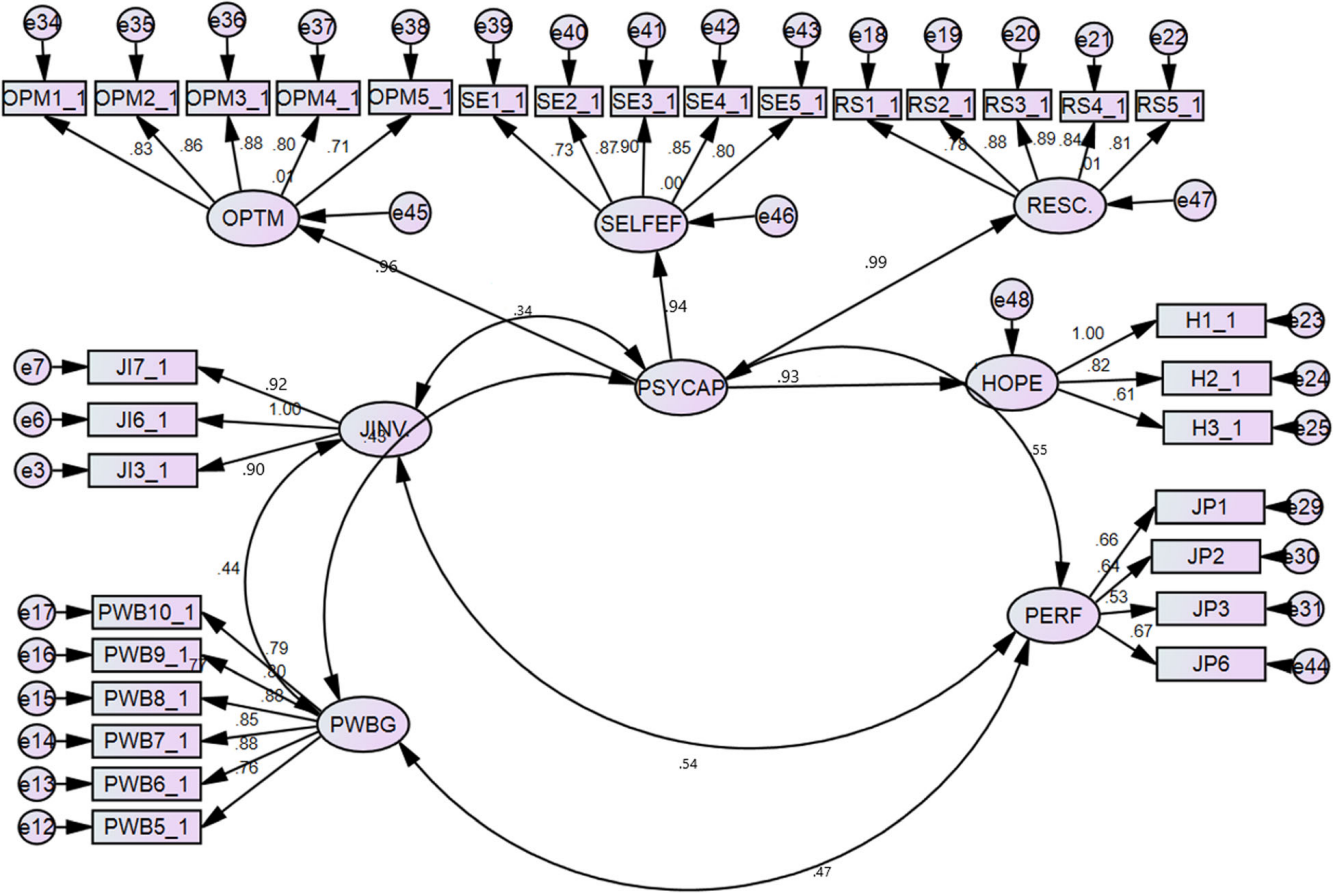
Second order confirmatory factor analysis of psychological capital and well-being, involvement, and performance

We sought to find out the relationship between the latent variables using AMOS version 22. The model consisted of the latent variables psychological wellbeing, job involvement, the moderating variable (psychological capital and its constructs i.e. optimism, self-efficacy, resilience, hope) and the dependent variable (performance sustenance) among family doctors in China. The items in the observed variables with low factor loadings were eliminated leaving the strong ones to be included. For instance, on hope, H4, H5 were eliminated, Psychological well-being PWB1, PWB2, PWB3 PWB4 were also eliminated. Performance sustenance JP4 and JP 5 which showed low loadings were also eliminated. Figure 2 shows the second-order confirmatory factor analysis of psychological capital and well-being, involvement, and performance. The fit indices for the model were, (χ2 = 141.284, df = 61, χ2/df = 2.316 TLI = .988, CFI = .982, NFI = .955, IFI = .993, RFI = .965 RMSEA = .035.

We tested the significance of psychological capital as a moderator between psychological well-being and job involvement. The model showed adequacy as an indication of a good fit for the data. The fit indices of the final measurement model were, χ2 = 245.654, df = 103 χ2/df = 2.384, CFI = .976, TLI = .982, RFI = .925, IFI = .966, and RMSEA = .050.

**Fig. 3:**
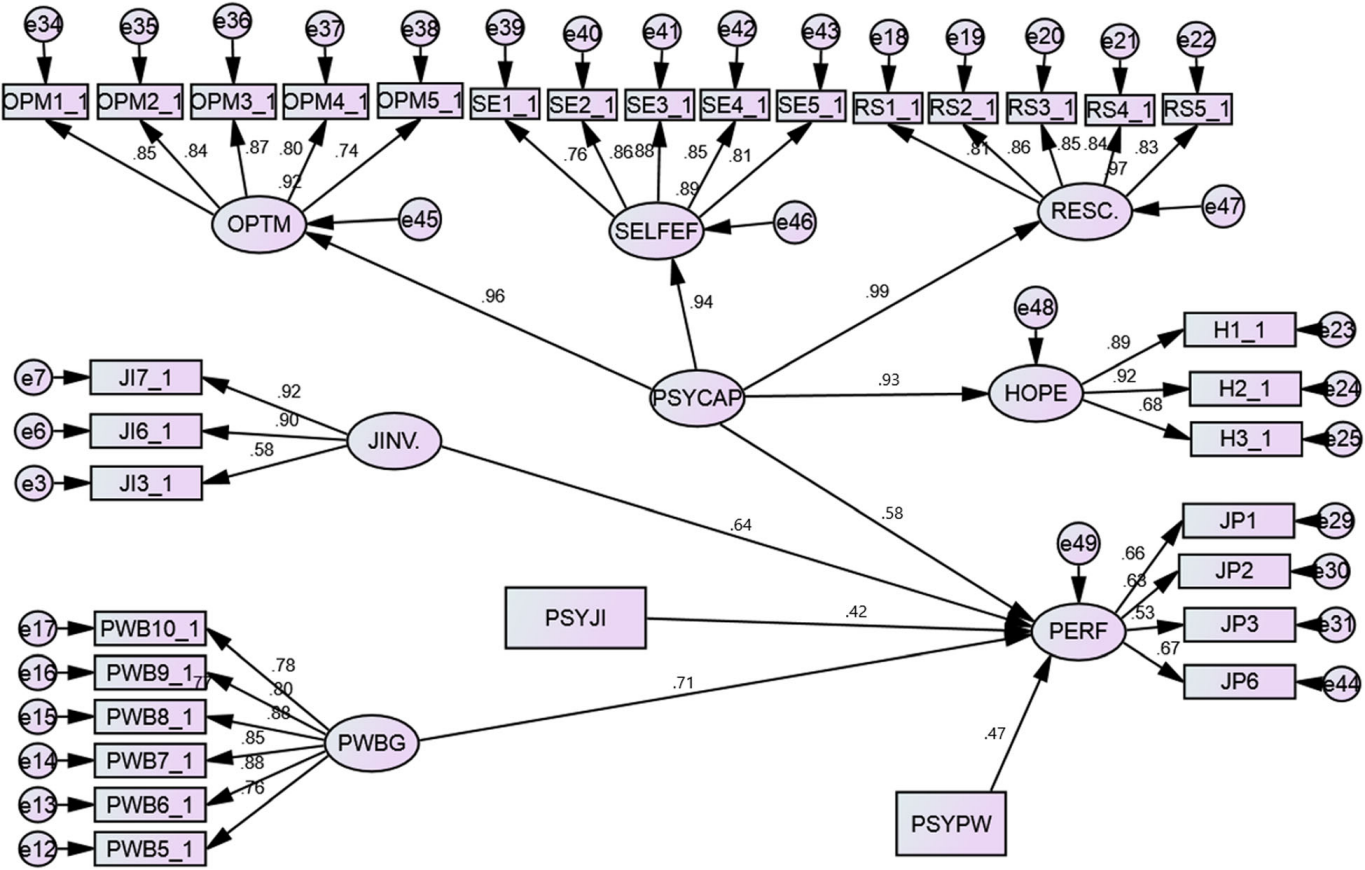
Path analysis of the moderated model of psychological capital, psychological well-being, job involvement and performance

The model confirmed H3a: that psychological well-being and job involvement directly influence performance sustenance of family doctors with PWB .71 (*p* > 0.5), Job Involvement .64(p > 0.5) and H3b: was confirmed .42 (p > 0.5) that psychological capital moderates significantly between job satisfaction and performance sustenance. Psychological capital interacts with both psychological well-being and job involvement to predict the performance sustenance of family doctors. Figure 3 shows the final path analysis of the moderated model of psychological wellbeing, job involvement, psychological capital and performance sustenance.

### Indirect effect of the moderator

We did a bootstrap of the 2000 sample to find out the indirect effect of the interaction, details are presented in Table 2

Table 3 shows the results of the path coefficient analysis of the structural equation model. A unit change in a family doctors’ psychological wellbeing influences performance positively by 0.74 and is statistically significant at a 95% confidence interval. Similarly, a change in job involvement causes a positive change in the performance of family doctors in the selected cities. The coefficient value of 0.64 and a *p*-value of 0.000 statistically justify this claim. The moderating effect of psychological capital on the relationship between family doctor’s psychological wellbeing and their performance is also positive. The coefficient is statistically significant at a 95% confidence interval. This is also the case with the moderating effect of psychological capital on the relationship between job involvement and performance. These cases support the assumption that psychological capital is a significant factor in stimulating positive mental, psychological and emotional behavioural outcomes among family doctors in China

**Table 3:** Indirect effect of the moderator

Parameter			Estimate	Lower	Upper	P
PERF	<−--	PWBG	0.71	5.552	39.439	.000
PERF	<−--	JINV	0.64	4.690	34.488	.000
PERF	<−--PsyCap<−--	PWBG	0.47	5.308	37.996	.000
PERF	<−-- PsyCap<−--	JINV.	0.42	−.052	.075	.009

** Correlation is significant at the 0.01 level (2-tailed) at the diagonal

* Correlation is significant at the 0.05 level (2-tailed)

## Discussions

China’s Healthy China 2030 Initiative has changed the view of the family practice profession from a mere gatekeeping practice to the pivot of overall primary healthcare delivery in China. Family doctors have assumed extended responsibility to manage risks and uncertainties, care for individuals in the context of their families and communities, and address the totality of patients. This underscores the ambition of China to train 750,000 more GPs in the next decade to consolidate its primary health gains. However, the results of this study suggest that endemic psychological and mental wellbeing concerns among family doctors may erode the gains already achieved. Traditionally, family doctors in China have considered themselves “people’s doctors” yet, a stressful workday, endless rules and regulations, poor wages and salaries are perceived dehumanizing routines attenuating the growth of family doctors and the primary healthcare system in China [17, 28].

These and many other hindrances have taken away a strong sense of the mutually beneficial long-term therapeutic relationship between family doctors and patients which is the central psychological factor for doctors’ willingness to accept family doctor jobs. The result of these challenges is that young doctors, in particular, see family doctor services as less prestigious and an unfulfilling arena to build a noble career [9]. The results of this study show that when psychological capital can interact with other job-related attitudes to generates positive and significant results thereby sustaining the performance of family doctors. Consequently, enhancing the well-being of health professionals such as the family doctor system, for instance, is a sure way of bettering the lives of the larger society. Similarly, a health employee who feels satisfied with the level of responsibilities and a positive work environment identifies with the organization by way of job involvement. In countries such as UK (where the family doctor system is well established), it contributes to solving nearly 90% of health-related problems and only rare or complex conditions are referred to specialists in secondary or tertiary hospitals. To ensure China reaps the benefits of the family doctor system and guarantee its sustainability, a strong and well-motivated family doctor workforce must be pursued. The results of this study support the need to build and develop family doctors that are mentally, psychologically and emotionally healthy.

To achieve the target of training and retaining 750,000 more GPs human resource managers in China’s health sector must focus on helping doctors overcome the challenges that negatively affect psychological and mental well-being. Some of these include high workload, avoidable bureaucratic requirements, and problems of low salary and limited recognition of their competency by the public, compared with hospital specialists. Being able to satisfy family doctors’ psychological needs can offer them the belief that they are masters of their own decisions, which when enabled, reflects in various job-related attitudes of satisfaction, peak performance, and increase in commitment levels, job involvement, and ultimately psychological well-being.

Theoretically, this study confirms the self-determination theory and further contributes to the theoretical outcomes of the development of self-determination theory and its application to supporting human resources for healthcare from an emerging country perspective. Precisely because this study extends the theory in practice by providing an additional outcome of sustained positive performance to the self-determination theory (SDT) aside from what the theorist propounded.

This is particularly relevant to the expansion of the self-determination theory discourse because the essence of developing individuals to be self-reliant and self-motivated in an organization or field of specialization is to ensure their commitment to the said organization based on strong mental, emotional and psychological wellbeing. In this regard, when employees are emotionally glued to an organization, it guarantees high-level performance because they begin to appreciate the fact that they can balance their personal goals and that of the organization. A condition that ensures a win-win situation enables employees to give off their best in the execution of tasks assigned to them. Most importantly, when employees such as family doctors are emotionally committed to an organization, they become intrinsically motivated. Intrinsic motivation is the foundation of the self-determination theory. An intrinsically motivated family doctor who has developed a more emotional attachment to the job will place the clients or patients ahead of any challenges faced in the line of duty. This provides additional validity of the confirmation of the self-determination theory especially among the healthcare professionals in China.

Additionally, family doctors and other health employees who develop affective commitment dedicate and consign themselves to making the work environment a better place. An effectively committed employee takes on additional responsibilities to enhance individual and organizational performance and becomes autonomous in motivating himself and others. Importantly, when a healthcare employee develops affective commitment, it makes an individual a team player in the task execution of the institution. In terms of the setting, our extension of the theory is relevant because we conducted our study in a different environment outside the areas the theory was conducted.

## Conclusion

This study intended to examine the relationship between job involvement, psychological well-being, and performance among family doctors in China. It again considered the influence of the interaction effect of psychological capital on psychological well-being, job involvement leading to performance sustenance on the family doctor system. The study confirmed all the hypotheses that job involvement correlates positively and significantly with performance sustenance while psychological well-being significantly correlates with performance sustenance,. Secondly, the interaction effect of psychological capital significantly affects the performance sustenance of healthcare employees positively. Health professionals such as family doctors experience a lot of pressure that weighs heavily on their psychological and emotional wellbeing. For this reason, a healthcare professional who experiences positive emotions affects the total behaviour which culminates in a performance. The results lay credence to preceding studies that observed that healthcare professionals who demonstrated subjective happy feelings brought on by the pleasure in the profession and work environment they appreciate shows in their performance. Additionally, health professionals who experience sentiments or emotions that give the impression of having purpose and meaning in the life they live translate it the task performance Family doctors who experience a sense of drive, happiness, and satisfaction identifies with the organization in terms of task execution, relationship with co-workers, and forming an identity with the organization they serve.. Again the higher-order construct of psychological capital relate significantly with job involvement to sustained performance. Psychological capital which consists of constructs such as hope, resilience, optimism, and self-efficacy reflects significantly on performance. This suggests that an upsurge in psychological capital equally leads to an improvement in psychological well-being. Similarly, an increase in the level of psychological capital leads to the job involvement of employees.

## Data Availability

The data for this research is held by the authors and will be made available upon reasonable request.

## Abbreviations

PsyCap: Psychological Capital
SDT: Self-Determination Theory
AVE: Average Variance Extracted
CFA: Confirmatory Factor Analysis
SE: Standard Error
CM: Covariance Matrix
SEM: Structural Equation Model
